# Failure to Improve Lumbar Lordosis After Single-Level TLIF for Degenerative Spondylolisthesis Does Not Impair Clinical Outcomes at 8.6 Years Postoperatively: A Prospective Cohort of 32 Patients

**DOI:** 10.3390/jcm14155457

**Published:** 2025-08-03

**Authors:** Klemen Bošnjak, Rok Vengust

**Affiliations:** 1 Department of Orthopaedic Surgery, University Medical Centre Ljubljana, 1000 Ljubljana, Slovenia; 2 Faculty of Medicine, University of Ljubljana, 1000 Ljubljana, Slovenia

**Keywords:** lumbar lordosis, degenerative spondylolisthesis, TLIF, sagittal balance, clinical outcomes

## Abstract

**Background**: We aimed to analyze radiographic sagittal balance parameters in patients who underwent a TLIF procedure for single-level degenerative spondylolisthesis with a mean follow-up of 8.6 years and to determine whether lumbar lordosis affects long-term clinical outcomes. **Methods**: This prospective study included 32 patients who underwent single-level TLIF surgery for degenerative spondylolisthesis. Radiographic analysis of sagittal balance parameters and clinical examination including Oswestry Disability Index (ODI) scores were performed preoperatively, postoperatively, and at the last follow-up. A minimal clinically important difference threshold of 30% was accepted as clinically relevant. **Results**: Mean postoperative lumbar lordosis (LL) and segmental lordosis (SL) failed to improve postoperatively; nevertheless significant improvements in short- and long-term postoperative ODI scores were demonstrated (*p* < 0.001). Thoracic kyphosis (TK) and global sagittal balance parameters shifted anteriorly after 8.6 years (*p* < 0.001), but this increase did not affect clinical outcomes. **Conclusions**: Adequate decompression and solid bone fusion are foremost required to achieve improved long-term clinical outcomes in single-level TLIF procedures. In our studied cohort, failure to improve lordosis did not impair clinical outcomes postoperatively. With aging, thoracic kyphosis and anterior malalignment increase, and after 8.6 years, clinical improvements are starting to become insignificant.

## 1. Introduction

Degenerative spondylolisthesis is a common spinal disease and mainly a consequence of physiological aging, although its exact pathomechanisms remain unclear [1,2]. Listhesis leading to local kyphosis is often associated with loss of lumbar lordosis (LL), leading to anterior spinal malalignment [1,2,3]. Deterioration of standing spinal balance can result in compensatory mechanism activation, leading to muscle fatigue, chronic back pain, and disability [2,3,4].

With poor quality of life and progressively worsening lower back pain, reconstructive spinal surgery is often justified [4]. In the past several years, concepts highlighting the importance of sagittal balance analysis in spinal fusion procedures have emerged [1,2,4]. Spinopelvic parameters are now widely recognized to affect clinical outcomes in long spinal fusion; however results regarding short-segment fusions are also emerging [5,6]. In a literature review and meta-analysis, postoperative lumbar lordosis–pelvic incidence (LL-PI) mismatch and failure to increase LL have been found as predictive factors for unfavorable outcomes [6,7]. Literature reports emphasize the importance of restoring LL in proportion to the pelvic incidence (PI) value to prevent fixed sagittal imbalance and chronic postoperative pain [2,4,8]. Using a regression model with 268 asymptomatic adults, Le Huec and Hasegawa proposed that postoperative LL should be approximated to calculated values of LL = 0.54 × PI + 27.6 [4]. However, in terms of long-term outcomes, radiographic parameters are not yet clearly connected with clinical outcome parameters [9].

To address the pathology of spondylolisthesis, TLIF is one among several fusion techniques [5,10,11]. To date, few studies have compared sagittal parameters and functional outcomes in single-level TLIF with 5–6 years of follow-up [5,6,9,11]. However, no comparable studies with longer-term evaluations have been published thus far. With the present study, we aimed to analyze radiographic sagittal balance parameters in patients who underwent a TLIF procedure for single-level degenerative spondylolisthesis with a mean follow-up of 8.6 years and to determine whether lumbar (LL) or segmental (SL) lordosis affects long-term clinical outcomes.

## 2. Materials and Methods

A total of 76 patients were assessed as eligible to be included in our study. After applying the exclusion criteria and the invitations to participate, 9 were excluded. The exclusion criteria were spondylolisthesis at more than one level, additional types of spinal coronal deformity with Cobb ˃ 10°, a history of additional spinal surgery, spinal inflammatory conditions, and flexion contractures of the hips or knees.

**Figure 1:**
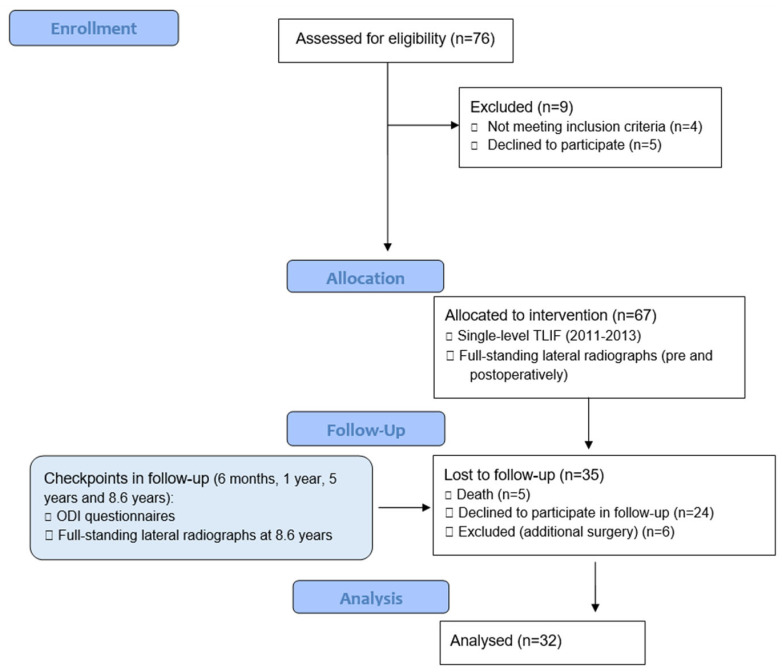
Flow diagram of the cohort model.

The TLIF procedure was performed between March 2011 and December 2013 at a single institution. A total of 67 patients underwent a single-level instrumented L3/4 or L4/5 TLIF for degenerative Meyerding grade I spondylolisthesis. The last follow-up recruitment was conducted from December 2019 to December 2022. Between the procedure and the last follow-up period, check-ups and questionnaires were applied at 6 months, 1 year and 5 years postoperatively. An additional 35 patients were lost during this follow-up period (Figure 1). The mean age of 19 female and 13 male participants at the last follow-up was 73.6 ± 8.5 years. At the time of the procedure, their average age was 65.0 ± 8.5 years. The mean follow-up period equaled to 103.4 ± 13.6 months, or 8.6 ± 1.1 years. The study was registered at ClinicalTrials.org on 20 November 2019, with the identifier NCT04174144.

### 2.1. Surgical Procedure

All patients were operated on by three senior spinal surgeons. In the knee–chest position, a single-level instrumented TLIF was performed using polyaxial pedicle screws (Xia 3^®^, Stryker, Portage, MI, USA) and a crescent-shaped interbody cage (T-Plus^®^, RTI Surgical, Alachua, FL, USA). Successful reduction was achieved in 62.5% (20/32). Decompression of the lateral recess and foramina was performed in case of significant stenosis. Osteotomy of the endplates was performed in anterior portions of endplates, followed by insertion of morselized bone mixed with a bone graft substitute. The interbody cage at its maximal feasible height was placed posterior to bone grafts. To complete the procedure, compression over the rods was performed.

### 2.2. Clinical and Radiographic Analyses

Patients were examined clinically preoperatively and postoperatively at 6 months, 1 year, 5 years, and 8.6 years. At the same checkpoints, ODI questionnaires were administered. As suggested in the literature, a minimal clinically important difference (MCID) threshold of 30% was accepted as a clinically relevant improvement of ODI scores [12,13].

**Figure 2:**
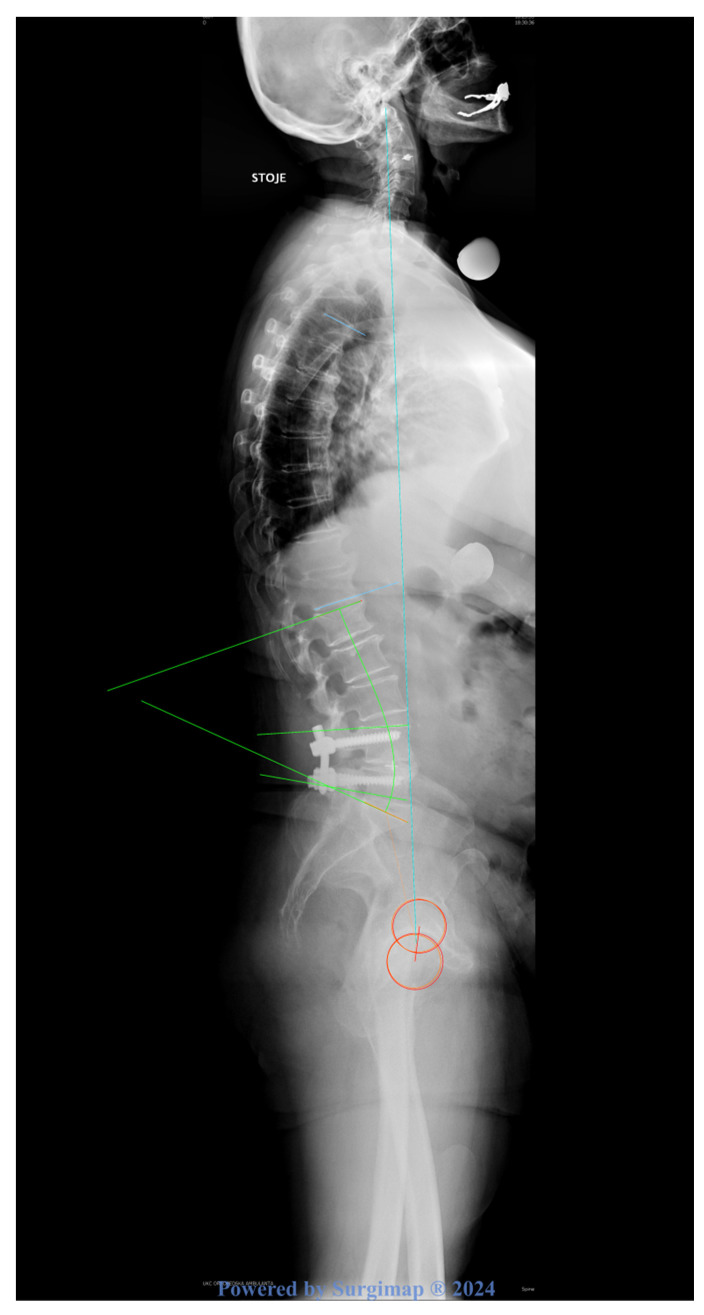
Example of measured parameters defined in Table 1, on full-standing lateral radiograph.

Radiographic parameters were analyzed preoperatively, within 1–2 days postoperatively, and at 8.6 years. Full-standing lateral radiographs as proposed by Morvan et al. [14] were used. Images were analyzed by both authors (Surgimap^®^, Nemaris Inc.™, New York, NY, USA). The parameters measured were lumbar lordosis (LL), segmental lordosis (SL), the odontoid–hip axis angle (OD-HA), thoracic kyphosis (TK), pelvic incidence (PI), pelvic tilt (PT), and sacral slope (SS) (Table 1 and Figure 2) [15,16]. The ideal LL was calculated according to the formula proposed by Le Huec and Hasegawa:

Ideal LL = 0.54 × PI + 27.6°(1)

The postoperative deficit of LL was defined as the difference between postoperative LL and the ideal LL (postoperative LL-ideal LL).

### 2.3. Statistical Analyses

Power analysis was performed before conducting this study. To detect a 30% improvement in minimal clinically important difference (MCID) in the ODI baseline score of 50 with a standard deviation of 15, a sample size of at least 32 patients would be required to achieve a power of 0.8. The power analysis of the correlation models was conducted separately. To achieve a power of 0.8, at least 31 patients needed to be included to detect an effect size of 0.35.

Statistical analysis was conducted using the SPSS for Windows 20.0 Software (SPSS Inc., Chicago, IL, USA). The one-way repeated measure ANOVA test with Bonferroni correction was used for the comparison of preoperative and postoperative values. A stepwise, multiple regression model was used for correlation examination. All statistical tests were significant at *p* < 0.05.

## 3. Results

### 3.1. Population Analysis

In our cohort, the ODI significantly improved postoperatively; the improvement was maintained throughout the 8.6 years postoperatively (*p* < 0.001) (Table 2). A postoperative improvement in ODI scores above the MCID threshold was maintained for 5 years; however it failed to reach the threshold at 8.6 years by 1.3 points. In our cohort the MCID resulted in an ODI improvement by 15 points, which is in accordance with the score (14.9) reported in the literature [12].

In our cohort LL and SL failed to improve postoperatively, as no significant differences between pre- and postoperative values were observed. Compared to their preoperative values, TK increased by 6.4°, and consequently, OD-HA increased by 6.5° in 8.6 years (Table 3). Normal values of OD-HA range from −5° to +2° [16], and mean values of 2.2° indicate borderline sagittal imbalance in our cohort at the latest follow-up. Radiographic signs of solid bone fusion were present in 93.7% (30/32) at the 1-year follow-up and in 100% at 8.6 years postoperatively.

### 3.2. Analysis of Correlations

The analysis of correlation revealed that LL at 8.6 years can be predictive of the ODI outcome at long-term follow-up; however no such correlation was found to be significant preoperatively and in the early postoperative period. However, the deficit of LL values revealed good correlations with the spinopelvic compensatory increase in PT and the decrease in TK preoperatively, postoperatively, and at 8.6 years. Correlations between OD-HA, TK, and clinical outcome parameters were not significant at any point in time.

Furthermore, a multiple regression analysis was performed, with the ODI as the primary dependent variable and the radiographic parameters listed in Table 3 as the independent variables. The results indicated that none of these parameters were statistically significant predictors of ODI values. The results of the correlation analyses are represented in Table 4, Table 5 and Table 6. The regression models are represented in Table 7.

## 4. Discussion

With the present study, marked long-term clinical improvement after single-level TLIF for degenerative spondylolisthesis was demonstrated. The results are comparable with studies reporting the 5–6-year clinical outcomes of short spinal fusion procedures [5,9,17,18]. Our study demonstrated the improvement in ODI scores culminating at 1 year postoperatively, then declining but nevertheless remaining statistically significant throughout the 8.6-year follow-up (*p* < 0.001). However, with the threshold of MCID in our cohort set to 30%, the mean 8.6-year ODI score failed to reach clinical significance due to a difference of 1.3 points.

Marked clinical improvement was achieved, despite failing to increase SL and LL. In the cohort studied, adequate decompression and solid bone fusion was achieved in 94% at the 1-year follow-up and 100% at the last follow-up. On the contrary, the mean SL declined by 0.8° postoperatively, while LL declined by 1.8° postoperatively and additionally by 1.2° after 8.6 years. Therefore, the total deficit of LL increased by 3° after 8.6 years. This can be attributed to the surgical technique, the knee–chest position, and not placing the cage anteriorly enough, achieving a reduction of only 62.5%. In contrast to our studied cohort, gains of SL ranging from +3.8 to +8.1° were reported when the cage was placed more anteriorly relative to the anulus [10,19,20,21]. Additional lordosis could also be gained by using a more lordotic-shaped cage and combining the resection of facet joints with rod compression techniques [11,20,21,22]. In principle, restoring SL enables the pelvis to rotate and allows PT and SS to return to their standard theoretical values, thereby reducing compensatory mechanisms that contribute to disability [2,3,4]. However, studies have shown that improving SL does not translate to improving the overall LL or spinal alignment [20,23]. Additional factors, such as reduced spinal stenosis, careful instrumentation with solid bone fusion, and multiple other biomechanical factors, play a more important role in determining the overall lordosis, spinal alignment, and long-term clinical outcomes postoperatively [18,21,22,23].

The observed lack of correlation between the lordotic and clinical outcome parameters in our cohort is consistent with findings from other mid- to long-term studies in the literature that report on clinical outcomes following single-level fusion procedures. In a meta-analysis of 13 studies with a mean follow-up ranging from 10 to 121 months, Rhee et al. found no significant correlation in sagittal balance improvement, lumbar lordosis restoration, and clinical outcomes for patients undergoing single-level lumbar fusion [18]. Tay et al. reported no significant differences in clinical outcomes between reduction and in situ fusion with postoperative lordosis decline for low-grade spondylolisthesis at the 5-year follow-up [17]. In the prospective study with a 6-year follow-up by Bredow et al., pelvic tilt with pelvic rotation and not lordosis predicted clinical outcomes after short-segment fusion surgery [9]. Our results indirectly support these findings, as the LL deficit of our participants negatively correlated with a compensatory increase in PT, but no direct correlations between outcome parameters and PT could be demonstrated in our cohort.

In the long term, factors beyond lordotic parameters may influence sagittal balance and the aging process of the spine. Over a period of 8.6 years, a significant decline in anterior sagittal alignment was observed in our population. We found a significant increase in OD-HA from −4.3° in a population aged 65.0 years to +2.2° in a population aged 73.6 years (*p* < 0.001), resulting in a borderline imbalance of OD-HA for this age group [16,24]. Given the 3° decline in lordosis observed in our cohort, these findings emphasize that the etiology of sagittal balance in elderly patients is multifactorial. In this context, Aoki et al. and Le Huec et al. reported on the dysfunction of supportive paravertebral tissues, such as disks, arthritic facet joints, and muscles, causing long-term posture imbalances [5,16]. Manakul et al. observed that spinal stenosis symptoms correlate with increased forward bending compensation and higher sagittal parameters, including SVA, PT, and PI-LL mismatch, which may be a result of functional adaptation to radicular pain [25]. Additionally, as the spine ages, erector muscles weaken, resulting in thoracic kyphosis and failure in maintaining a normal standing position [16]. In these circumstances, muscle energy is expended to maintain body posture and movement, resulting in long-term pain, fatigue, and disability [4,26]. Furthermore, Yang et al. reported that TK may serve as an indicator of sagittal imbalance and disability in elderly populations [27]. In our cohort, the mean TK increased by 6.4° after 8.6 years, which is similar to that by Ferrero et at., who reported significantly increasing thoracal kyphosis values of 60.3° in an elderly sagittal population with degenerative spondylolisthesis [1]. Their mean age value of 73.4 years additionally corresponds to the mean age of our cohort at the latest follow-up of 73.6 years. In the setting of sagittal imbalance of the aging spine with increasing thoracic kyphosis, single-level corrections of LL cannot result in a significant improvement in balance [17,18,27]. At this stage, larger and multi-level procedures are required to address these changes.

### Limitations

When performing the analysis of sagittal balance, a comparative analysis by dividing patients into groups according to PI is important [2]; however due to the limited numbers of participants, such an analysis would be underpowered. Moreover, of the initially eligible 76 patients, only 32 were available for the final analysis, resulting in a 58% attrition rate. This raises concerns about attrition bias. Without comparing those who completed follow-up to those lost, it is unclear if the final sample represents the original cohort. For instance, patients with poorer outcomes may have been more likely to drop out, potentially leading to an overestimation of the surgical procedure’s effectiveness. Longer-term evaluations with a higher number of participants, control groups, and minimal follow-up loss should pose further insight into the natural history of post-fusion lumbar spines and remain to be a subject of further studies.

## 5. Conclusions

Adequate decompression and solid bone fusion are foremost required to achieve improved long-term clinical outcomes in single-level TLIF procedures. In our study cohort, failure to improve lordosis did not impair clinical outcomes postoperatively. However, the absence of clinical impairment does not necessarily indicate optimal sagittal alignment or long-term biomechanical health. With aging, thoracic kyphosis and anterior malalignment increase, and after 8.6 years, clinical improvements are starting to become insignificant.

## Figures and Tables

**Table 1:** Measured parameters as proposed by Duval-Beaupère et al. and Le Huec et al. [15,16].

Parameter	Measurement Procedure
Pelvic incidence (PI)	The angle between the line perpendicular to the center of the upper S1 endplate and the line connecting this point to the axis of the femoral heads.
Segmental lordosis (SL)	The angle between the superior endplate of the superior vertebrae and the inferior endplate of the inferior vertebrae of the two fused vertebral bodies.
Lumbar lordosis (LL)	The angle between the superior endplate of L1 and the superior endplate of S1.
Pelvic tilt (PT)	The angle between the vertical line and the line connecting the center of the sacral endplate to the axis of the femoral heads.
Sacral slope (SS)	The angle between the horizontal line and the line tangent to the upper S1 endplate.
Thoracic kyphosis (TK)	The angle between the superior endplate of Th4 and inferior endplate Th12.
Odontoid–hip axis angle (OD-HA)	The angle between the vertical line and the line connecting the highest point of the odontoid process (dens) to the axis of the femoral heads.

**Table 2:** Comparison of clinical outcome parameters (mean values ± SD).

	Preoperatively	6 Months	1 Year	5 Years	8.6 Years	*p* Value
**Mean ODI ± SD**	49.5 ± 15.1	31.5 ± 16.4	28.5 ± 17.6	33.0 ± 15.2	35.8 ± 15.3	<0.001 *

* Statistically significant values at *p* < 0.05.

**Table 3:** Comparison of radiographic parameters (mean values ± SD).

	Preoperatively	Postoperatively	8.6 Years	*p* Value
PI	56.1 ± 10.5	56.0 ± 10.6	56.1 ±1 0.4	0.635
LL	50.6 ± 11.4	48.8 ± 8.9	47.6 ± 8.8	0.118
SL	19.9 ± 5.6	19.1 ± 5.6	19.4 ± 5.6	0.337
Deficit of LL	−7.2 ± 10.1	−9.0 ± 8.0	−10.2 ± 8.4	0.093
PT	23.9 ± 7.2	24.2 ± 7.3	21.1 ± 7.4	0.007 *
SS	32.2 ± 9.1	31.9 ± 8.8	34.8 ± 8.6	0.013 *
TK	35.7 ± 1.9	34.3 ± 1.9	42.1 ± 1.7	0.001 *
OD-HA	−4.3 ± 3.4	−2.8 ± 3.8	2.2 ± 4.4	0.001 *

* Statistically significant values at *p* < 0.05.

**Table 4:** Results of the correlation analysis between parameters preoperatively (R value and *p* value).

	ODI	PI	LL	SL	Deficit of LL	PT	SS	TK	OD-HA
ODI	1								
PI	R = −0.194 *p* = 0.144	1							
LL	R = −0.313 *p* = 0.040 *	R = 0.475 *p* = 0.003 *	1						
SL	R = −0.061 *p* = 0.371	R = 0.444 *p* = 0.005 *	R = 0.536 *p* = 0.001 *	1					
Deficit of LL	R = −0.242 *p* = 0.091	R = −0.025 *p* = 0.445	R = 0.867 *p* < 0.001 *	R = 0.356 *p* = 0.023 *	1				
PT	R = −0.065 *p* = 0.362	R = 0.510 *p* = 0.001 *	R = −0.236 *p* = 0.097	R = −0.084 *p* = 0.324	R = −0.554 *p* = 0.001 *	1			
SS	R = −0.171 *p* = 0.175	R = 0.735 *p* < 0.001 *	R = 0.726 *p* < 0.001 *	R = 0.564 *p* < 0.001 *	R = 0.408 *p* = 0.01 *	R = −0.206 *p* = 0.129	1		
TK	R = −0.304 *p* = 0.045 *	R = −0.117 *p* = 0.262	R = 0.417 *p* = 0.009 *	R = −0.029 *p* = 0.437	R = 0.541 *p* = 0.001 *	R = −0.211 *p* = 0.123	R = 0.035 *p* = 0.425	1	
OD-HA	R = 0.203 *p* = 0.133	R = 0.129 *p* = 0.241	R = −0.277 *p* = 0.062	R = −0.053 *p* = 0.387	R = −0.386 *p* = 0.015 *	R = −0.129 *p* = 0.241	R = 0.254 *p* = 0.081	R = −0.054 *p* = 0.385	1

* Statistically significant values at *p* < 0.05.

**Table 5:** Results of the correlation analysis between parameters postoperatively (R value and *p* value).

	ODI	PI	LL	SL	Deficit of LL	PT	SS	TK	OD-HA
ODI	1								
PI	R = −0.270 *p* = 0.068	1							
LL	R = −0.107 *p* = 0.280	R = 0.492 *p* = 0.002 *	1						
SL	R = −0.053 *p* = 0.387	R = 0.356 *p* = 0.023 *	R = 0.306 *p* = 0.044 *	1					
Deficit of LL	R = 0.072 *p* = 0.348	R = −0.157 *p* = 0.195	R = 0.782 *p* < 0.001 *	R = 0.087 *p* = 0.318	1				
PT	R = 0.069 *p* = 0.353	R = 0.528 *p* = 0.001 *	R = −0.191 *p* = 0.148	R = 0.175 *p* = 0.169	R = −0.596 *p* < 0.001 *	1			
SS	R = −0.385 *p* = 0.015 *	R = 0.752 *p* < 0.001 *	R = 0.714 *p* < 0.001 *	R = 0.270 *p* = 0.067	R = 0.273 *p* = 0.065	R = −0.160 *p* = 0.191	1		
TK	R = 0.120 *p* = 0.256	R = −0.236 *p* = 0.097	R = 0.277 *p* = 0.062	R = −0.120 *p* = 0.257	R = 0.483 *p* = 0.003 *	R = −0.047 *p* = 0.4	R = −0.247 *p* = 0.087	1	
OD-HA	R = −0.132 *p* = 0.236	R = 0.193 *p* = 0.145	R = 0.032 *p* = 0.430	R = −0.09 *p* = 0.313	R = −0.096 *p* = 0.301	R = −0.186 *p* = 0.155	R = 0.378 *p* = 0.016 *	R = 0.051 *p* = 0.391	1

* Statistically significant values at *p* < 0.05.

**Table 6:** Results of the correlation analysis between parameters after 8.6 years (R value and *p* value).

	ODI	PI	LL	SL	Deficit of LL	PT	SS	TK	OD-HA
ODI	1								
PI	R = −0.272 *p* = 0.066	1							
LL	R = −0.507 *p* = 0.002 *	R = 0.402 *p* = 0.011 *	1						
SL	R = −0.014 *p* = 0.470	R = 0.319 *p* = 0.038 *	R = 0.184 *p* = 0.157	1					
Deficit of LL	R = −0.354 *p* = 0.023 *	R = −0.25 *p* = 0.084	R = 0.785 *p* < 0.001 *	R = 0.356 *p* = 0.023 *	1				
PT	R = 0.06 *p* = 0.372	R = 0.575 *p* < 0.001 *	R = −0.297 *p* = 0.049 *	R = −0.084 *p* = 0.324	R = −0.701 *p* < 0.001 *	1			
SS	R = −0.374 *p* = 0.017 *	R = 0.717 *p* < 0.001 *	R = 0.741 *p* < 0.001 *	R = 0.564 *p* < 0.001 *	R = 0.298 *p* = 0.049 *	R = −0.157 *p* = 0.195	1		
TK	R = −0.059 *p* = 0.374	R = −0.241 *p* = 0.092	R = 0.252 *p* = 0.082	R = −0.029 *p* = 0.437	R = 0.435 *p* = 0.006 *	R = −0.163 *p* = 0.186	R = −0.153 *p* = 0.201	1	
OD-HA	R = −0.026 *p* = 0.444	R = 0.156 *p* = 0.197	R = −0.277 *p* = 0.062	R = −0.176 *p* = 0.168	R = −0.289 *p* = 0.054	R = −0.025 *p* = 0.445	R = 0.197 *p* = 0.140	R = 0.199 *p* = 0.258	1

* Statistically significant values at *p* < 0.05.

**Table 7:** Results of multiple regression analysis.

Regression Model	R	R^2^	Std. Error of the Estimate	F	Model Significance
Preoperatively	0.479	0.229	15.323	0.856	0.566
Postoperatively	0.622	0.386	16.033	1.810	0.127
8.6 years after procedure	0.604	0.365	14.153	1.649	0.165

## Data Availability

The original contributions presented in this study are included in the article. Further inquiries can be directed to the corresponding author.

## Abbreviations

The following abbreviations are used in this manuscript:

TLIF	Transforaminal lumbar interbody fusion
ODI	Oswestry Disability Index
MCID	Minimal clinically important difference
LL	Lumbar lordosis
SL	Segmental lordosis
TK	Thoracic kyphosis
PI	Pelvic incidence
PT	Pelvic tilt
SS	Sacral slope
OD-HA	Odontoid–hip axis angle
